# Case report: Complete response of recurrent locally advanced basal cell carcinoma following addition of vismodegib to neoadjuvant cemiplimab therapy

**DOI:** 10.3389/fonc.2025.1500785

**Published:** 2025-01-30

**Authors:** Chalette Lambert-Swainston, Wolfram Samlowski, Brandon Reynolds, Amin Hedayat

**Affiliations:** ^1^ Kirk Kerkorian School of Medicine, Department of Internal Medicine, University of Nevada Las Vegas (UNLV), Las Vegas, NV, United States; ^2^ Comprehensive Cancer Centers of Nevada, Las Vegas, NV, United States; ^3^ Department of Internal Medicine, University of Nevada School of Medicine, Reno, NV, United States; ^4^ Reynolds Plastic Surgery, Las Vegas, NV, United States; ^5^ Quest Diagnostics, Associated Pathologists Chartered, Las Vegas, NV, United States; ^6^ American Melanoma Institute, Las Vegas, NV, United States

**Keywords:** hedgehog inhibitor, checkpoint inhibitor, targeted therapy, immunotherapy, keratinocyte carcinoma

## Abstract

Locally advanced basal cell carcinoma (laBCC) remains a significant management challenge. Historically, surgery or radiotherapy represented the major treatment options. Recently, hedgehog inhibitors and checkpoint inhibitors have demonstrated significant activity. The optimal sequencing of these approaches is not yet known. We describe a case of laBCC that recurred after definitive radiotherapy. This patient was treated with neoadjuvant cemiplimab with minimal response. Cautious addition of vismodegib with ongoing cemiplimab treatment was well tolerated and resulted in significant tumor regression. A wide excision demonstrated a pathologic complete response. Further evaluation of cemiplimab and hedgehog inhibitor combination therapy in la BCC appears warranted.

## Background

Basal cell carcinoma (BCC) represents the most common form of skin cancer in the United States, with 3.3-5.4 million individuals affected per year (1). A small percentage of patients neglect a developing BCC, which can become large and deeply invasive. The frequency of locally advanced basal cell (laBCC) carcinoma is not well characterized but is estimated to be 1.28/100,000 individuals in the US each year, resulting in 7940 annual cases (2). Historically, radical surgery or radiotherapy were the major treatment options for laBCC patients. Unfortunately, these treatments resulted in a high recurrence rate.

The “hedgehog” signaling pathway was identified as a key regulatory pathway for epidermal cell growth and development (3). Mutations in this pathway occur in the majority of patients with either sporadic or inherited forms of BCC (e.g., Gorlin’s syndrome). These mutations lead to constitutive activation of the pathway, resulting in progressive cell growth (4). Oral small-molecule hedgehog inhibitors (HHI), such as vismodegib and sonidegib have significant activity in la BCC and can induced rapid and deep responses in many patients with modest systemic toxicity (5, 6). Unfortunately, most patients eventually progress despite continued HHI therapy (7, 8).

The PD-1 antibody cemiplimab has also been evaluated in patients with laBCC, who were not candidates for curative surgery or radiation, and who had progressed after HHI therapy. These studies demonstrated that cemiplimab has a significant clinical response rate in HHI refractory patients, with about 1/3 of patients achieving long-term responses or remissions (9). More recently, neoadjuvant cemiplimab has been tested prior to definitive local therapy in keratinocyte-derived skin cancers (10).

## Case report

**Figure 1:**
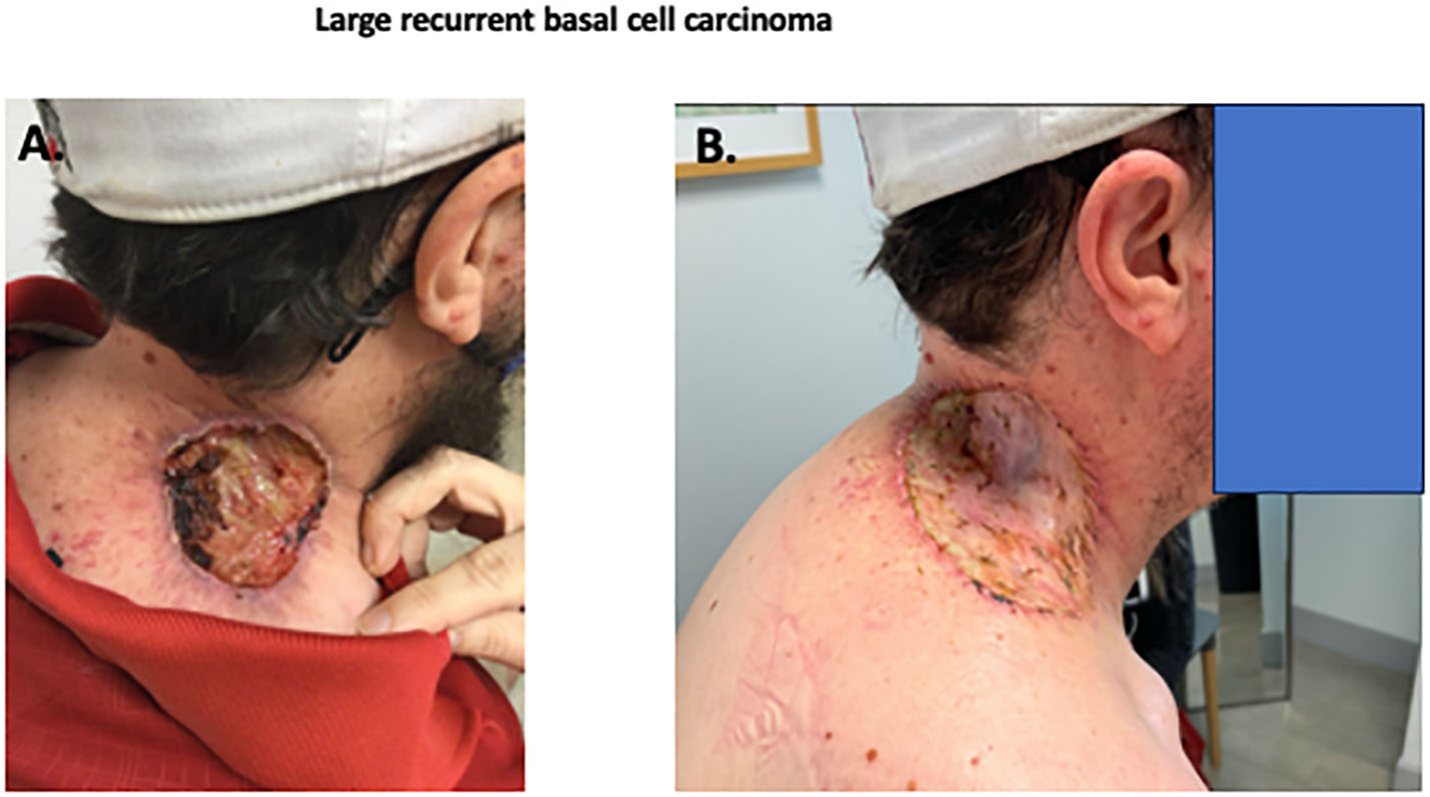
Photographs of recurrent BCC. Initial skin lesion **(A)** demonstrating a deeply invasive nodular basal cell carcinoma, Resection of skin lesion with skin graft **(B)** with no residual BCC.

A 43-year-old Caucasian man presented with a large 8 x 8 cm ulcerated basal cell carcinoma located at the base of the neck overlying the right trapezius (**Figure 1A**). The patient had been diagnosed with laBCC four years previously that had been treated with external beam radiotherapy with 73.8 cGy in 40 fractions. The patient was lost to follow-up for two years, but eventually sought medical attention due to the rapidly increasing size to 8x5 cm of a recurrent lesion, associated with pain and bleeding.

**Figure 2:**
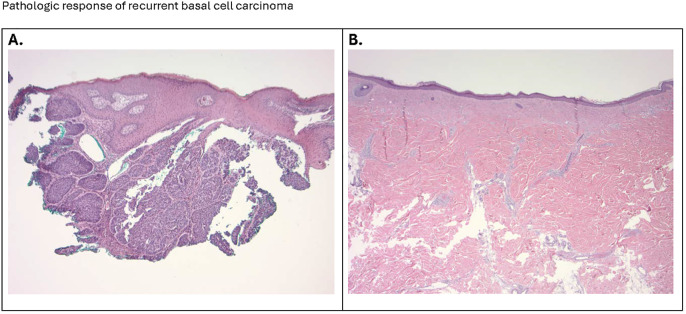
**(A)** The micrograph (100x) shows a nodular basal cell presenting as islands and nodules of basaloid cells with a peripheral palisading arrangement, embedded in a fibrous stroma. A noticeable cleft or retraction artifact separates the tumor islands from the surrounding stroma. The dermis shows a sparse inflammatory infiltrate. **(B)** The photomicrograph (20x) confirms the successful surgical removal of the site of the original basal cell carcinoma, with clear margins and satisfactory wound healing. The histological section illustrates a region of dense fibrous connective tissue, indicative of scar formation, with no evidence of residual basaloid cell nests. Collagen bundles are tightly packed and aligned parallel to the skin surface, characteristic of a reparative process.

As the patient had previously received maximal radiotherapy, a surgical consultant suggested a wide surgical excision. Due to size of the lesion, there were concerns about potential deep tissue invasion and proximity to cranial nerve XI. A large skin graft or flap reconstruction were considered necessary as part of the post-operative reconstruction. Due to his perception of potential side-effects, the patient declined prolonged hedgehog inhibitor therapy. After multidisciplinary discussion the patient was treated with neoadjuvant cemiplimab therapy, with the goal of cytoreduction. After three doses of cemiplimab (350mg fixed dose) given intravenously three weeks apart, there was little clinical change in the lesion (8 x 5 cm). At the time of the third dose, oral vismodegib was cautiously added (150 mg p.o. daily), to try to increase the clinical response. After this modification of treatment, the lesion decreased in size and began to re-epithelialize. The patient received a total of 6 doses of cemiplimab (350 mg every 3 weeks) and 11 weeks of daily oral vismodegib (150 mg/d), with a progressive reduction in size of the lesion to approximately 2-2.5 cm. The patient subsequently underwent excision of the previously involved area with a skin graft repair (**Figure 1B**). Evaluation of the resection specimen by pathology confirmed a complete remission (**Figure 2**).

**Figure 3:**
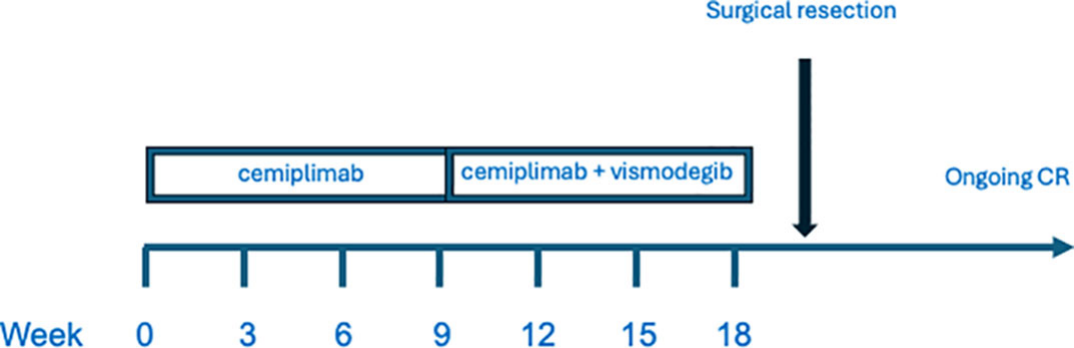
Treatment schema.

Vismodegib treatment was well tolerated with side effects. The patient described minimal loss of taste and mild hair thinning, which resolved after drug discontinuation. There was no apparent cemiplimab associated immune adverse events, either during monotherapy or following addition of vismodegib. The patient currently continues to be disease free at 24 months post resection. A graphic schema to the treatment course is provided (**Figure 3**).

## Discussion

We evaluated a young man with a large laBCC that had recurred after primary radiotherapy. Multidisciplinary discussion recommended initial systemic therapy.

We considered the use of HHI therapy. While this treatment has been shown to have a high response rate, eventual progression appeared likely. Vismodegib has produced objective response rates of 43-68.5% (7, 11), with a median duration of response of 26.2 months in laBCC (7). Sonidegib has produced an objective response rate of 56% in la BCC, with a median duration of response of 26.1 months (8). In addition, these clinical trials reported high rates of adverse events, resulting in frequent treatment discontinuation.

We therefore initiated cemiplimab therapy. Cemiplimab was initially tested in advanced BCC as salvage therapy after initial surgery, radiotherapy and HHI therapy. A phase II study in 84 laBCC patients described a response rate of 32% in patients who had previously failed or were intolerant HHI therapy with an adverse event rate of 20.4% (9). Based on this phase II study, cemiplimab was subsequently approved by the US Food and Drug Administration (FDA) in 2021 for patients with locally advanced basal cell carcinoma (laBCC) previously treated with a hedgehog pathway inhibitor (HHI) or for whom a HHI is not appropriate.

Several reports have suggested significant clinical activity of cemiplimab and other PD-1 antibodies as a neoadjuvant therapy for locally advanced cutaneous squamous cell carcinoma (10). There is less data concerning neoadjuvant PD-1 antibody therapy in laBCC. One recent case series has demonstrated successful treatment of advanced BCC with neoadjuvant PD-1 inhibitors (12). A significant percentage of durable complete pathological responses was reported. The theoretical advantage of neoadjuvant therapy is potential for tumor cytoreduction, reducing the scope of necessary surgery. Individualized response assessment is possible, allowing subsequent tailored therapy. It appears likely that neoadjuvant therapy also results in a stronger and broader T cell response.

After two cycles of treatment, it became apparent that our patient had a very minimal response to cemiplimab. We considered cautious addition of HHI treatment based on three case reports that suggested clinical activity of this combination (13–15). In addition, recent data suggested that HHI may decrease immunosuppressive features of tumor microenvironment, in a manner that may potentiate checkpoint inhibitor treatment (16). The combination of cemiplimab therapy with vismodegib was well tolerated. The patient described minimal loss of taste and mild hair thinning, which resolved after drug discontinuation. This combined treatment induced substantial tumor regression. Complete surgical resection of the affected skin (avoiding deeper tissue resection) demonstrated a pathologic complete response. This unmaintained response has continued for 24 months after treatment discontinuation.

In conclusion, this case demonstrates a novel treatment option for the locally advanced BCC. This patient did not respond to cemiplimab alone. The addition of HHI to PD-1 therapy led to a marked reduction in the locally advanced tumor. There was no apparent added toxicity from combination therapy. Upon resection, it was determined that the patient had achieved a pathologic complete remission. This case suggests a rationale to further evaluate cemiplimab with HHI to improve pathologic responses and curability of large laBCC lesions.

## Data Availability

The original contributions presented in the study are included in the article/supplementary material, further inquiries can be directed to the corresponding author/s.
